# Transferability of interventions in health education: a review

**DOI:** 10.1186/1471-2458-12-497

**Published:** 2012-07-02

**Authors:** Linda Cambon, Laetitia Minary, Valery Ridde, François Alla

**Affiliations:** 1 EA 4360 Apemac, Faculté de médecine, Université de Lorraine, 54250, Vandoeuvre-lès-Nancy, France; 2 Inserm, CIC-EC, Centre hospitalier universitaire, 54000, Nancy, France; 3 Department of Social and Preventive Medicine, CRCHUM, 3875 Saint-Urbain, Montreal, QC, H2W 1 V1, Canada; 4 Université de Lorraine, Faculté de Médecine, Ecole de Santé Publique, 9 avenue de la Forêt de Haye – BP 184, F-54505, Vandœuvre-lès-Nancy, France

**Keywords:** Transferability, Applicability, Health education, Health promotion, Evidence-based, Evaluation, Assessment, Complex intervention

## Abstract

**Background:**

Health education interventions are generally complex. Their outcomes result from both the intervention itself and the context for which they are developed. Thus, when an intervention carried out in one context is reproduced in another, its transferability can be questionable. We performed a literature review to analyze the concept of transferability in the health education field.

**Methods:**

Articles included were published between 2000 and 2010 that addressed the notion of transferability of interventions in health education. Articles were analyzed using a standardized grid based on four items: 1) terminology used; 2) factors that influenced transferability; 3) capacity of the research and evaluation designs to assess transferability; and 4) tools and criteria available to assess transferability.

**Results:**

43 articles met the inclusion criteria. Only 13 of them used the exact term “transferability” and one article gave an explicit definition: the extent to which the measured effectiveness of an applicable intervention could be achieved in another setting. Moreover, this concept was neither clearly used nor distinguished from others, such as applicability. We highlight the levels of influence of transferability and their associated factors, as well as the limitations of research methods in their ability to produce transferable conclusions.

**Conclusions:**

We have tried to clarify the concept by defining it along three lines that may constitute areas for future research: factors influencing transferability, research methods to produce transferable data, and development of criteria to assess transferability. We conclude this review with three propositions: 1) a conceptual clarification of transferability, especially with reference to other terms used; 2) avenues for developing knowledge on this concept and analyzing the transferability of interventions; and 3) in relation to research, avenues for developing better evaluation methods for assessing the transferability of interventions.

## Background

Health education aims to give people the skills they need to adopt and maintain positive health behaviours. It combines personal and collective intervention strategies to develop the knowledge and competencies required to take better decisions related to health. This process is generally part of a health promotion approach that includes other strategies for modifying the environment and orienting health services more toward prevention [1]. Health education interventions are complex interventions that combine several complexity factors [2]. As well, the outcomes of these interventions result both from the interventions themselves and from the context for which they are developed [3]. So, a key question raised by these interventions has to do with their transferability, which has been defined as the extent to which the measured effectiveness of an applicable intervention could be achieved in another setting [3]. This issue of transferability is a major limitation in the use of research results by health stakeholders and decision-makers, and thus in the process of evidence-based health education and promotion [4]. Yet, in this field, there is a real issue around promoting the development of evidence-based health policies [5-8], in that they need to align responses to local needs with the development of effective actions.

But how is transferability defined, evaluated, and taken into account in the health education field?

To our knowledge, and despite its importance, this issue has been poorly studied in health education, in contrast to other health sectors, such as health policy and healthcare [9-11].

We therefore reviewed published articles based on four research questions: 1) What is the terminology used to describe the concept of transferability? 2) What are the factors that influence transferability? 3) Do research and evaluation designs make it possible to assess transferability? 4) What tools and criteria are available to assess transferability?

## Methods

### Identification and selection of articles

We searched MEDLINE via PubMed and SCOPUS databases for articles. We chose those databases because they provide the most thorough coverage in the health education field [12].

The selection criteria were as follows:

· articles;

· published between January 2000 to last searched date (May 2010);

· in French or English;

· addressed the concept of transferability defined, even implicitly, as the extent to which the measured effectiveness of an applicable intervention could be achieved in another setting [3];

· concerned health education interventions [13]

We defined a list of keywords using semantic progressive steps, expanding the search to terms proposed in the Medical Subject Heading Terms’ (MESH) terminology framework: *Translation, Diffusion, Dissemination, External validity, Adaptation, Generalization, Generalizability*. We also searched for articles with the keywords [“transferability” OR “generalizability” OR “generalization” OR “translation” OR “diffusion” OR “dissemination” OR “external validity” OR “adaptation”] AND [“health promotion” OR “public health” OR “health education”] AND [intervention OR program].

We selected articles by reading the titles and abstracts and, if necessary, the full text.

### Content analysis

The full text of the selected articles was analyzed using a specifically developed grid that explored the four research questions. The articles were independently analyzed by two of the authors (LC, FA). In cases of disagreement, the readers performed a third reading together.

We followed the PRISMA checklist [14] in carrying out the study and preparing the manuscript.

## Results

### Selected articles

The search identified 3,143 abstracts. We excluded 3,100 abstracts because they:

· did not relate to a health education intervention (indeed, we chose “public health” and “health promotion” as keywords to ensure retrieval of all abstracts addressing health education) (1,139 articles)

· addressed the transfer of knowledge, skills, and practices, in particular in abstracts retrieved with the keywords “dissemination” and “diffusion” (797 articles).

**Figure 1:**
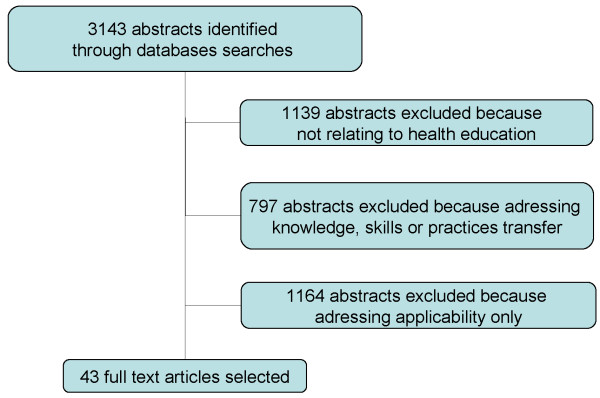
3143 abstracts identified through database searches.

· addressed applicability only, in particular in articles retrieved with the keywords “adaptation”, “dissemination”, “translation”, and “generalization” (1,164 articles).Finally, 43 abstracts met the selection criteria (i.e. Figure 1**Flow Diagram**). [3,15-56]Of the 43 articles retained, we distinguished three types:

22 theoretical and methodological articles presenting analyses of the concept of transferability or related topics, such as the evaluation of interventions, the external validity of studies, or the process of adapting and implementing interventions within an evidence-based perspective;

14 describing one intervention, either a primary intervention or an adaptation of an experimental intervention in a different setting;

7 literature reviews that mainly addressed transferability in terms of generalizing an intervention.

Table 1 describes the articles (i.e. Table 1).

**Table 1:** Description of selected articles

**Authors**	**Year**	**Using transferability term**	**Types of articles**	**Theme**	**Detail**
**Zubrick**[15]	2005	yes	intervention	mental health	provides adaptation modalities
**Belansky**[16]	2006	yes	intervention	nutrition and physical activity	provides adaptation modalities
**Frijiling**[48]	2003	no	intervention	cardiovascular diseases	efficacy studies
**Tsey**[18]	2005	yes	intervention	global health	provides adaptation modalities
**Glasgow**[26]	2004	no	theoretical and/or methodological	all themes	about REAIM model/tool
**Roush**[17]	2009	yes	theoretical and/or methodological	all themes	about RCT models and transferability factors
**Rychetnik**[19]	2002	yes	theoretical and/or methodological	all themes	about transferability factors and quality of evidence
**Wang**[3]	2006	yes	theoretical and/or methodological	all themes	about limits of RCT model and transferability factors
**Heller**[56]	2008	yes	theoretical and/or methodological	all themes	about external validity
**Zeicmeister**[41]	2008	no	theoretical and/or methodological	mental health	about limits of RCT model and need for qualitative evaluation
**Blackstock**[49]	2007	no	intervention	BPCO	efficacy studies
**Gray**[50]	2000	no	intervention	alcohol	efficacy studies
**Malterud 2001**[20]	2001	yes	theoretical and/or methodological	all themes	about qualitative studies
**Elford**[21]	2003	yes	theoretical and/or methodological	HIV	external validity, limits of RCT models, and transferability factors
**Nielsen**[51]	2008	no	intervention	nutrition	efficacy studies
**Glasgow**[33]	2003	no	intervention	diabetes	about process evaluation and use of RE-AIM model
**Hautmann**[52]	2008	no	intervention	mental health	provides adaptation modalities
**Flowers**[22]	2002	yes	intervention	HIV	efficacy studies
**Cattan**[23]	2005	yes	review	loneliness	assesses the transferability of several studies
**Victora**[25]	2004	no	theoretical and/or methodological	all themes	type of studies, limits of RCT models, dose-intervention and dose response
**Estabrooks**[28]	2003	no	theoretical and/or methodological	physical activity	focuses on a tool to assess external validity : RE-AIM model/tool
**Baranowski**[31]	2000	no	review	nutrition	analysis of intervention process, about qualitative evaluation
**Buijs**[32]	2003	no	intervention	global health and seniors	analysis of intervention process, about qualitative evaluation
**Glasgow**[27]	2003	no	theoretical and/or methodological	all themes	about RE-AIM model and contextual factors
**Glasgow**[55]	2006	yes	theoretical and/or methodological	all themes	about RE-AIM model and contextual factors
**Spoth**[34]	2008	no	theoretical and/or methodological	global health, teenagers	evidence-based public health and translational research
**Klesges**[35]	2008	no	review	obesity	efficacy studies
**Reinschmidt**[40]	2010	no	review	diabetes	accounting for adaptations of an experimental study
**Lorig**[53]	2004	no	intervention	patient education	provides adaptation modalities
**Perrin**[45]	2006	no	intervention	patient education	providing adaptation modalities
**Kwak**[36]	2005	no	theoretical and/or methodological	global health	transferability factors, notably focused on participation rate
**Cohen**[29]	2008	no	theoretical and/or methodological	global health	focuses on the RE-AIM model, types of adaptation, the need to drive evaluation in real settings
**Card**[46]	2009	no	theoretical and/or methodological	HIV	describes adaptation process in seven steps
**Feldstein**[37]	2008	no	theoretical and/or methodological	all themes	describes PRISM model to assess external validity
**Bull**[38]	2003	no	review	all themes	uses RE-AIM model
**Chen**[30]	2009	no	theoretical and/or methodological	all themes	limits of Campbellian model and RCT model
**Stevens**[24]	2001	yes	review	mental health	provides adaptation modalities
**Cuijpers**[47]	2005	no	theoretical and/or methodological	all themes	transferability factors
**Mukoma**[44]	2009	no	intervention	HIV	process intervention
**Eakin**[39]	2002	no	review	obesity	uses RE-AIM model
**Rimer**[42]	2001	no	theoretical and/or methodological	all themes	evidence-based public health, limits of RCT models
**Dzewaltowski**[54]	2004	no	theoretical and/or methodological	physical activity	describes interest of using REAIM model
**Dzewaltowski**[43]	2004	no	theoretical and/or methodological	all themes	describes interest of using RE-AIM model

### The terminology used to describe the concept of transferability

Only 13 articles [3,15-24,55,56] used the precise term “transferability” or a derivative of the term (“transferable”). Only one article [3] gave a detailed definition of transferability. However, some terms were used as synonyms for transferability (by order of frequency: “dissemination” [14 articles], “external validity” [13 articles], “generalization” [11 articles], “generalizability” [7 articles], “adaptation” [7 articles], “translation” [3 articles], “diffusion” [1 article], “translatability” [1 article], and “applicability” [1 article]).

Some articles referred to the notion of “pure” transferability (outcomes-focused) or did not discriminate between the concepts of transferability and applicability (i.e., the extent to which an intervention process could be implemented in another setting [3]). The terms most often associated with transferability were “generalizability” and “external validity”, although they have different meanings. We will come back to the distinction between these terms and the concept of transferability later in this article.

### Factors influencing transferability

Schematically, two levels of influence on transferability were described [25] : indirect (outcomes are not transferable because the terms and conditions for implementing the intervention are different) or direct (for the same implementation modalities, different outcomes are obtained) (i.e. Table 2).

**Table 2:** Factors influencing transferability

**Type of influence**	**Types of factors**
**Indirect influence “dose intervention” factors**	· whether the professionals followed the experimental protocol
	· the group size
	· the existence of incentives for the beneficiaries to facilitate and support their participation
	· the training and coaching of participants in the protocol’s implementation
	· the modifications for the new context
**Direct influence “dose response factors”**.	· category 1: Factors present in the target population that reduce the extent to which the intervention affects the outcome, defined as "*antagonism*."
	· category 2: Factors present in the target population that enhance the extent to which the intervention affects the outcome, defined as "*synergism*".
	· category 3: This category determines the beneficiaries’ actual need with respect to the intervention. This concept is based on the theory that the same dose will have less effect if there is less need for it and is defined as a "*curvilinear dose–response association*."
	· category 4: The presence or absence of interventions that are antagonistic to the studied intervention, for example, the presence of messages dissonant to that conveyed by the intervention.
	· category 5: The absence of a necessary cofactor in the causal chain of the intervention.
	· category 6: The presence or absence of an external intervention that is synergistic with the objective of the intervention studied.

#### Indirect influence

Implementation modalities and the conditions under which an intervention is executed have an impact on the outcomes [26,54,55]; these elements are thus transferability factors. The following factors were highlighted: whether the professionals followed the experimental protocol; the group size; the existence of incentives to facilitate and support beneficiaries’ participation; training and coaching of the participants in the protocol’s implementation; and, possibly, the modifications required for the new context. By extension to the field of clinical research, the concept of delivery of the intervention was called the "dose intervention" [25]. This concept refers to a qualitative and quantitative assessment, including implementation terms and beneficiary participation. This notion was analyzed by the difference between efficacy and effectiveness studies in 11 theoretical and methodological articles [3,19,20,25,27-30,45,54,55] and one intervention-based article [15] that showed how effectiveness could differ when a clinical practice was extended into primary care. One of these articles especially highlighted the influence of methods of recruitment, of training the professionals, and of maintaining their competencies [45].

The results of effectiveness studies performed in conditions closer to the "real world" were more transferable. In particular, Victora et al. [25] specified the parameters of dose-intervention variability and associated each of them with a specific type of efficacy or effectiveness study (i.e., clinical efficacy trial, public health regimen efficacy studies, public health delivery efficacy studies, public health program efficacy studies, and public health program effectiveness studies). Dzewaltowski [54] went even further, in modelling a drastic loss of effectiveness when modifying certain factors in a program on physical activity: the training of the professionals; the implementation of a routine with no required mobilization; adherence of practitioners; changes in competencies and in the implementation conditions. In this example, the effectiveness of the program, measured based on the participation of the beneficiaries, fell from the reference value of 100% in the initial program, to 0.4%.

#### Direct influence

Beyond the dose intervention issue, which explains much of the effect of variations in generalization, Victora et al. also pointed out the variability in an intervention’s effect even with identical implementation [25]. This level of influence was defined as the "dose response". This dose response may depend on the characteristics of the population and/or on the presence of environmental factors, both of which influence results independently of intervention modalities. These factors were classified into six categories.

Category 1 describes factors present in the target population that reduce the extent to which the intervention affects the outcome, defined as "*antagonism*." The factor may, for example, be about health education, or a passive event that generated mistrust, or a cognitive dissonance [57] of the beneficiary in relation to the intervention. Thus, specific interventions will have a positive impact on some subjects and a negative impact on others, depending on those people’s history, the representations they have of health issues, or even the method used in the intervention.

Category 2 describes factors present in the target population that enhance the extent to which the intervention affects the outcome, defined as "*synergism*". The factor may also be a passive but potentializing event, contrary to the previous example, that allows the beneficiary to pass, for example, from a Prochaska stage [58] to another behavioural change stage (i.e., the intervention will only work on subjects already sensitized, that is, ready to change).

Category 3 determines the beneficiaries’ actual need with respect to the intervention. This concept is based on the theory that the same dose will have less effect if there is less need for it, and is defined as a "*curvilinear dose–response association*." Health education practitioners in particular must pay special attention to emerging needs and representations before the intervention, either to adapt their action to them or to raise awareness of these sometimes unconscious needs and thus potentiate the effectiveness of the intervention.

Category 4 relates to the presence or absence of interventions that are antagonistic to the studied intervention, for example, the presence of messages dissonant from that conveyed by the intervention.

Category 5 relates to the absence of a necessary cofactor in the intervention’s causal chain. This category represents cases of important determinants of health-related behaviour, such as the inaccessibility of condoms despite information on the importance of their use.

Category 6 relates to the presence or absence of an external intervention that is synergistic with the objective of the intervention studied. One example would be a causal conflict generated by a nutritional intervention conducted in schools on pupils whose food balance at home is also undergoing change because their parents are on a diet. Determining what produces the outcomes—the school-based action, parental behaviour, or both—would be difficult.

### Ratings and assessments of transferability

Of the 43 articles, 18 specifically addressed the question of studies’ validity by emphasizing their internal and external validity; these included 12 theoretical and methodological articles [25-30,34,36,37,43,54,55], 2 intervention-based articles [32,33] and 4 literature reviews [31,35,38,39] (i.e. Table 3)*.* Internal validity is what makes it possible to conclude there is a causal relationship between the intervention and the outcome [25]. For internal validity of research, the randomized controlled trial is promoted as the standard. External validity, or generalizability, represents the measure of the extent to which the findings can be generalized to a wider population [59]. It allows the researcher to draw conclusions about the generalizability of the intervention. For this reason, there has been increased focus on the issue of external validity and greater recognition of this issue in selecting articles for publication [60]. The usual assumption is that the representativeness of the sample of individuals selected in the primary study normally ensures generalizability of the intervention to a larger population or, with some adaptation of the intervention, to a different setting [61], with the understanding that effective generalization is not always possible. This is the case only within the framework of a simple causal-chain intervention, for which the previously observed influence factors are not taken into account or are given little consideration. It might not be the case for health-related behaviours or, consequently, for health education [3,25,29-33,37,39,54,55]. Thus, the external validity of a study allows for conclusions on its “potential transferability” (is the intervention potentially generalizable?) by means of a reporting logic. Transferability is different from external validity. It is a process performed by the readers of research—particularly those involved in public health—in a logical analysis related to a specific setting [62] (would the measured effectiveness be identical to the primary intervention in this particular setting?). In addition, the question of external validity raises the question of appropriate assessment methods for ensuring transferability. In the Campbellian validity model, the stronger the internal validity of a study, the weaker the external validity, and vice versa [30]. Therefore, we could contrast the randomized controlled trial, with strong internal validity and weak external validity, and the observational study, with strong external validity and weak internal validity, taking into account all the intermediate stages, such as, particularly, in quasi-experimental studies.

**Table 3:** Ratings and assessments of transferability

**Topic studied**	**Number of articles**
Specifically addressed the question of studies’ validity by emphasizing their internal and external validity	18 articles : [25-30,34,36,37,43,54,55], 2 intervention-based articles [32,33] and 4 literature reviews [31,35,38,39]*.*
Limitations of generalizability of intervention in health education	11 articles [3,25,29-33,37,39,54,55]
Limitations of experimental frameworks for research in the health education field .	8 articles : 7 theoretical and methodological articles [3,17,19,21,25,30,41] and one intervention-based article [15]
The value of qualitative assessments that make it possible to explore and report on possible interactions among populations, interventions, and context and, therefore, to explain the outcomes	16 articles : 14 theoretical and methodological articles [3,17,19-21,25-27,29-31,41,42,54] and 3 intervention-based articles [14,32,33]

This contrast of studies raises the question of the usefulness of the randomized controlled trial for producing transferable outcomes in health education. Moreover, of the 43 articles, 7 theoretical and methodological articles [3,17,19,21,25,30,41] and one intervention-based article [15], addressed the limitations of experimental frameworks for research, agreeing on two observations: at the level of proof, the randomized controlled trial is the highest-rated evaluation method in terms of demonstrating causality [19] in a given context but raises many questions when trials are used in health promotion. Indeed, the trial is not always applicable in the field of health education for technical or ethical reasons, because of difficulties associated with selecting individuals to implement the interventions and controlling all variables that influence the results, as we have seen previously. These variables are specific to the beneficiaries, to their environment, and to the collective interactions between individuals. For these reasons, some authors consider observational and quasi-experimental studies to be the most feasible, acceptable, and/or appropriate study designs for evaluating public health interventions [19]. Furthermore, their experimental nature often limits interventions in terms of methodological aspects such as an oversimplified intervention context, being away from the real world, small sample size, and long-term outcomes not analyzed [21,41]. Finally, the principle of having a precise protocol for assessment and intervention appears to influence the outcomes [15,25] by moderating the dose intervention or dose response. Elford’s article [21] highlighted, in the field of HIV, limitations to the generalization of interventions that had been shown to be effective in an experimental context, when it came to reproducing the same results after transfer. Roush [17] stressed that randomization allows for a balanced distribution of factors involved in the causal intervention/outcomes ratio. Therefore, it is a key element of the internal validity of studies and helps reduce the assessment of antagonistic or synergistic aspects of these factors, whose importance we highlighted earlier, and therefore, of the transferability.

On the question of the randomized controlled trial, two perspectives could be distinguished. Zubrick [15], Rychetnick[19] and Wang [3] agreed that health promotion requires measuring effectiveness more than efficacy, and they called for reconsidering the methods, focusing more on experimental and quasi-experimental studies and observations. Victora et al. [25] meanwhile, moved away from discussions for or against controlled randomized trials, inviting researchers, instead, to consider choosing a study based on what they really want to obtain. Thus, the authors defined several study categories:

· Seeking an outcome that would be considered a probability assessment (i.e., did the program have an effect?) calls for a randomized controlled trial.

· Seeking an outcome that would be considered a plausibility assessment (i.e., did the program seem to have an effect above and beyond other external influences?) calls for observational studies with a control group (quasi-experimental).

· Seeking an outcome that would be considered an adequacy assessment (i.e., did the expected changes occur?) calls for an observational study.

Finally, 16 of the 43 articles highlighted the value of qualitative assessments that make it possible to explore and report on possible interactions among populations, interventions, and context and, therefore, to explain outcomes; these included 14 theoretical and methodological articles [3,17,19-21,25-27,29-31,41,42,54] and 3 intervention-based articles [14,32,33]. This is what is proposed in the realistic model [63]. However, the authors acknowledge that these methods, complementary to the randomized controlled trial, make it possible to identify, but not to demonstrate, the influence of various factors on the outcomes. Therefore, once the factors are identified, their influence could be shown, if possible, with randomized controlled trials [17]. Moreover, evaluation of the intervention’s implementation process is highlighted as providing necessary information to help explain "how it works" as well as to demonstrate "what works" [21,31,32,53]. Indeed, for lay health worker programs, the wider inclusion of qualitative research with the trials would have allowed us to explore a number of factors that might have influenced program outcomes. These include factors associated with the program itself, such as how the lay health workers were selected and trained and their relationship with communities and with the professional health workers, but also with the broader context of the program, such as political, social, or cultural conditions [64].

From this analysis, we can see that the gold standard methods—in particular, the randomized controlled trial—are not useful for assessing the transferability of results in health education. Alternative methods, qualitative approaches, and process evaluations are required to produce transferable knowledge. Thus, the evidence-based health education and promotion approach should focus on different modes of complementary or integrative studies, as in mixed-method evaluations [65], combining qualitative and quantitative methods. It also requires not only describing the outcome of an intervention (what works?), but also how it came to be (how does it work?).

### Tools and criteria available to assess transferability

Of the 43 articles, 6 theoretical and methodological articles [27,28,37,43,54,55] and one intervention-based article [33] discussed two tools for assessing the external validity of health promotion studies: RE-AIM (Reach, Effectiveness [or Efficacy, according to the study], Adoption, Implementation, and Maintenance) and the Practical, Robust Implementation and Sustainability Model (PRISM). No article proposed a framework or tool for assessing transferability.

The seven articles agreed that the criteria for internal validity may have been accurately reported in the studies, notably strengthened by the CONSORT (Consolidated Standards of Reporting Trials), but that this was not the case for criteria relating to external validity [26,28,30-32,34-39,54]. Nonetheless, the authors offered some frameworks for the analysis of external validity of health promotion studies.

The first of these frameworks is RE-AIM, which makes it possible to take into account, besides the efficacy or effectiveness assessment, the participation rate and representativeness of settings, the consistency with which different intervention components are delivered, the long-term outcomes on beneficiaries, and whether an innovation or program is retained or becomes institutionalized [26,33]. This model was promoted on the completion of studies and also in the production of a literature review to compare studies based on multiple and identical dimensions [26,28,35,38,39,54]. The literature reviews conducted using the RE-AIM model showed that very often data on all these dimensions was missing [35,36,38,39,54]. These authors highlighted that modulation of these variables considerably modified the impact of the intervention [55].

The second of these frameworks, based on implementation and thus referring more to applicability, is the PRISM model, which evaluates how health care programs or interventions interact with recipients to influence program adoption, implementation, maintenance, reach, and effectiveness. The model particularly facilitates [37] the diffusion of innovation by analyzing key factors for a program’s successful implementation and sustainability. Indeed, using key questions, this framework highlights elements associated with the success of an intervention’s implementation and sustainability in the RE-AIM key domains: the program (intervention), the external environment, the implementation and sustainability infrastructure, and the recipients. Assessing each key domain and its success factors early in the implementation effort is helpful to guide any necessary modifications. The authors believe further research is needed to determine whether the number of PRISM domains activated is an important predictor of success in other implementation and dissemination reports and which PRISM elements are most important for particular settings and clinical targets. Actually, the tool is intended more for translating research into practice than for assessing external validity.

### Transferability factors or types of factors

Of the 43 articles, 20 explicitly provided, as criteria for external validity, evaluation, or processes to adapt existing interventions, elements that could be used to build a typology of transferability factors. A first cornerstone is based on the RE-AIM framework [26-28,35,38,39,54]. A second cornerstone is based on a study of intervention processes and/or of the adaptation of interventions as sources for understanding the efficiency factors. A first group of authors [31,32,44] described how the assessment process helps to explain applicability and/or transferability. These process elements become potential categories of transferability factors. A second group of authors [40,45] examined not the intervention process, but the adaptation process. Unlike dose intervention, which modulates the intervention without fundamentally changing it, program adaptation is defined [46] by a process of change to reduce the dissonance between the characteristics and the new setting in which the program is implemented. This concept refers to the definition of adaptation criteria [40] and to the stages of this adaptation process that some authors have modeled [46]. These criteria or adaptation factors could, again, be categories or potential transferability factors.

Six articles—4 conceptual articles [3,19,21,47] and 2 intervention-based articles [15,18]—give specific examples of criteria beyond the categories. From these elements, we have structured a potential list of transferability factors or categories (i.e. Table 4).

**Table 4:** Categories of transferability factors

**Categorization of factors**	**Sub-categories or examples of factors**	**Source authors**
**Factors related to population**	Factors related to the representativeness and characteristics of the target population (Reach RE-AIM): age, ethnicity, socioeconomic status, income, health status	Glasgow 2004, Estabrooks 2003, Glasgow 2003, Klesges 2008, Bull 2003, Eakin 2002, Dzewaltowski 2004, Elford 2003; Wang, 2006; Cuijpers 2005; Rychetnik, 2002;
	Factors related to participation of the population (*Adoption* of RE-AIM): perceived benefits, incentive group, a positive atmosphere within the program, the program seen as a priority	Glasgow 2004, Estabrooks 2003, Glasgow 2003, Klesges 2008, Bull 2003, Eakin 2002, Dzewaltowski 2004, Zubrick, 2005; Buijs 2003
	Volunteerism and the autonomy of participants	Buijs 2003
	Cultural factors related to lifestyles and worldviews	Reinschmidt 2010, Rychetnik, 2002; Elford 2003;
	Cognitive factors depending on the age of recipients and their language, literacy, educational achievement	Reinschmidt 2010, Wang, 2006; Rychetnik 2002, Elford 2003
	Affective - motivational factors related to gender, ethnicity, religion and socioeconomic level	Reinschmidt 2010
**Factors related to the implementation**	Factors associated with all the resources and practices required to implement the intervention, including the cost and duration (*Implementation* of RE-AIM)	Glasgow 2004, Estabrooks 2003, Glasgow 2003, Klesges 2008, Bull 2003, Eakin 2002, Dzewaltowski 2004, Zubrick, 2005; Wang, 2005; Elford 2003
	Availability of resources for routine application of the intervention	
	Adaptability to the characteristics of the population	Tsey, 2005
	Adaptability of the program to local realities	Buijs 2003, Tsey 2005; Elford 2003
	"Comfort,” that is, an optimal intervention environment	Buijs 2003
	Mobilization methods that could vary depending on the characteristics of beneficiaries	Perrin 2006
	Compensation for the participation of professionals and beneficiaries	Perrin 2006
	Language used appropriate to the culture and origin of participants	Perrin 2006
	Accessibility of the intervention	Zubrick, 2005; Rychetnick, 2002; Elford 2003
	Relevance of the intervention to influence the risk factor and/or problem	Zubrick, 2005
	Feasibility of the intervention	Zubrick, 2005 : Elford 2003;
	Acceptability of the intervention	Zubrick, 2005; Wang, 2005; Elford 2003;
	Factors related to intervention: its model, its development, its delivery	Rychetnick 2002
**Factors related to professionals**	Providing all required instructions and intervention materials	Mukoma 2009, Cuijpers 2005
	A participatory training that takes into account the professionals’ diverse views and experiences and targets their attitudes, skills and self-efficacy to implement the intervention	Mukoma 2009, Perrin 2006, Cuijpers 2005,
	Involving professionals in developing and piloting the lessons, and reviewing the research instruments, skills.	Mukoma 2009, Wang, 2006; Rychetnick 2002
	Interest earned from the program by professionals in terms of their practice	Cuijpers 2005
	Enjoyment of the professionals	Buijs 2003
**Factors related to the environment**	Environmental factors related to the systemic dimension of the community	Reinschmidt 2010
	Recognition of unique institutional settings	Perrin 2006
	Factors related to politico-social context (health system, financing, services or existing alternative program, etc.).	Rychetnick 2002, Wang, 2006; Cuijpers 2005, Wang, 2006;
	Factors associated with interaction between the intervention and context	Rychetnick 2002
**Factors related to a specific health problem**	Prevalence of health problem in the population	Zubrick, 2005; Wang, 2005
	Prevalence of risk factors for the targeted health problem	Zubrick, 2005
	Convincing causal link between the risk factor that is the target of the intervention and the health problem	Zubrick, 2005
	Relevance of the problem statement to be treated by professionals (expert agreement)	Cuijpers 2005

## Discussion

Because of the complexity of health education interventions, especially the interaction between setting, intervention and outcome, the question of transferability is crucial when advocating evidence-based approaches. To understand this issue of transferability in health education, we conducted a review and analyzed 43 articles. The terms used to express the notion of transferability were varied, and, conversely, the term transferability was sometimes used to express another concept (generally applicability)*.* This initial analysis showed that this concept, resulting from the convergence of disciplines and the representations of each author, is only beginning to be defined and shared in this field.

We identified two levels of influence of transferability: dose intervention and dose response. The six categories of dose–response factors, in addition to those modulating dose intervention, show how the issue of transferability is complex, in that it can be influenced in two ways: either indirectly, through the implementation of the intervention, or directly, in terms of the beneficiaries’ response to the intervention, each being capable of reacting, as we have seen, differently from the other. Therefore, in health education, because it touches on the complex phenomena that behaviours represent, the result can totally escape the health stakeholders, regardless of the rigour with which they implement an intervention. In addition, some factors may act at both levels. For example, participants’ cognitive consonance with the message conveyed by the action might affect their participation (indirect effect, because if participants do not take action, they will not adhere to the message) or their health behaviour directly (they participated in the action but did not change the behaviour). These factors are known determinants of health behaviours, but unfortunately have not been considered operationally from the perspective of transferability.

The evaluation methods also play a role in the transferability of the data produced, especially if they refer to the gold standard in research. Indeed, with respect to the transferability of health education activities, the randomized controlled trial is now considered to have many limitations related to its applicability to the strictness of the protocol, which confers internal validity as well as the generalizability of routine processes, and its inability to make readable the interactions between the intervention, the environment and the population. It must therefore be enriched by other types of evaluation.

Chen questioned the Campbellian validity model that promotes the primacy of the trial and a research rule from the study of efficacy to the study of effectiveness and then to dissemination [30]. Applying this model, called the top-down approach, impedes the translation of research into practice in the public health field. Chen based his argument on two assumptions: 1) the effectiveness study is often ignored in favour of a direct transfer from the study of efficacy to dissemination; and 2) interventions designed from the experimental perspective can only rarely be established, adopted, and maintained in real conditions and routine organizations. So rather than taking note of these difficulties and trying, as did the RE-AIM authors, to promote the collection of maximal data to facilitate implementation of the Campbellian model, Chen questioned the logic itself. Accordingly, he introduced a complementary notion, “viable validity”, which he defined as the extent to which an intervention program is viable in the real world based on the characteristics of the intervention (i.e., it evaluates whether the intervention can recruit and/or retain ordinary people and be adequately implemented by ordinary implementers). He suggested an alternative model, which he defined as an “integrative validity model,” that corresponds better to the expectations of the professionals, because only an intervention recognized as viable can be evaluated on its effectiveness.

From this analysis, we can suggest that the current research model based on the primacy of internal validity does not allow for the production of transferable data in health education. However, alternative assessment methods, and the ongoing work on defining external validity, will help change it. This issue is not specific to health promotion, but rather it concerns more generally the so-called “complex interventions”, whose evaluation requires a combination of methods using different designs [2,66,67]. Thus, if we want stakeholders to base their interventions on evidence and effectiveness in different settings, we must address the following:

The promotion and development of more qualitative research, and better understanding of complex phenomena in any kind of health education to allow practitioners to clearly identify what created the outcomes, and whether they depend on the nature of the intervention, the dose intervention, or on the dose response. This process can only be achieved by expanding and recognizing other, complementary methods of research evaluation.

The development of tools to evaluate, from the practitioners’ perspective, an intervention’s transferability, given the large number of factors likely to influence it. On this last point, our goal was to clarify the concept in health education and to objectify it. The list contains all factors that may contribute to the development of this type of analysis tool for transferability, including a guide for adapting transferability as needed, depending on the existing factors.

### Methodological strengths and limitations

Even though the aim of this study was not to be comprehensive, it does have limitations related to the search strategy. In particular, articles were selected on the basis of abstracts. We may have missed articles that addressed the issue of transferability without it being mentioned in the abstract. Nonetheless, the consistency of the authors’ findings and the ease with which we were able ultimately to define a consensual list of factors among those debated by the authors argues that additional articles would not have contributed further to our findings.

As well, this review does not take into account other strategies to promote health—health public policy, supportive environments, health services reorientations—that pertain to other sectors of intervention. Indeed, it focuses on educational strategies for health promotion.

Finally, we relied particularly on the external validity criteria provided by the selected articles in the field of health promotion. However, there are other tools for assessing external validity in other intervention areas that contribute to evidence-based public health. These tools could be used, as we did with RE-AIM, to extrapolate transferability factors. However, we wanted to focus, as a first step, on an analysis of the concept in the specific field of health education. Undoubtedly, with further, more comprehensive work on the consolidation of a tool mentioned above, additional analysis of these tools would be necessary, as would the incorporation of this analysis of the transferability of planning frameworks (e.g., PRISM [37] PRECEDE/PROCEED [68]).

## Conclusions

In this review, we can suggest that the issue of transferability of interventions in health education is diffused within assessment research. Although transferability is a fuzzy concept, we tried to make it concrete by defining it along three lines, which are and could be investigated with further research: factors influencing transferability, research methods to produce transferable data, and the development of validated criteria to assess the transferability of health education interventions. That being said, based on this analysis, and in order to advance the question of transferability in health education, we can already formulate the following propositions:

First, this summary helps to clarify the following conceptual definitions: the term transferability should be used when assessing the results of an intervention in relation to its original experimental trial. In contrast to external validity, which is a researcher’s perspective on the generalizability of his action, transferability assumes the view of the health professional, who transfers an action that has been evaluated elsewhere into his own specific context. Finally, when speaking about envisioning the conditions for implementing it in another context, the term applicability is the most appropriate (i.e.).

### Terms

Concept from the researcher’s point of view:

Generalizability: the extent to which the findings can potentially be generalized to an unspecified or wider population [59].

External validity: characteristic of the studies which provides the basis for generalizability to other populations, settings, and times [69].

Concept from the point of view of the readers/users of research:

Transferability: the extent to which the measured effectiveness of an applicable intervention could be achieved in another setting [3].

Applicability: the extent to which an intervention process could be implemented in another setting [3].

Secondly, with respect to knowledge development, the concept of transferability has been barely objectified. We were able to identify the presentation of some criteria, but the criteria we extracted and analyzed seemed more or less accurate, specific and structured, in that they were often produced by validity or process assessment or adapted from an intervention. How they were developed was not always shown and appeared to be the result of both common sense and exchange among practitioners involved in an intervention, rather than of any methodical and rigorous process. From the known elements of the issue, a transferability criteria tool could be developed that could be used to assess the transferability of interventions by comparing the settings of research studies with the setting in which the practitioner must implement an intervention. This guide could be used to incorporate transferability criteria into the reporting of studies, thus making the research more transferable and therefore more useful to health stakeholders. Such a tool would inform decision-makers and health stakeholders in choosing a specific intervention in a particular setting or in performing the necessary and possible adjustments to achieve real efficiency. This tool would thus contribute to the implementation of evidence-based health practice [11].

Finally, with respect to avenues for further research, this review highlighted the efforts that must be made to develop research in this field that will be more easily transferable and more useful for health stakeholders. Evaluation methods should also be developed that could take into account the dimension of transferability as well as internal validity. This will probably require the development, in research, of combinations of studies or approaches for assessing complex interventions [70].

## Competing interests

The authors declare they have no competing interests**.**

## Authors’ contributions

LC and FA conceived the study, analyzed and interpreted the data, and drafted the paper. LM and VR participated in the interpretation of the data and in the drafting. All authors read and approved the final manuscript.

## Pre-publication history

The pre-publication history for this paper can be accessed here:

http://www.biomedcentral.com/1471-2458/12/497/prepub
